# The Mad Genius Stereotype: Still Alive and Well

**DOI:** 10.3389/fpsyg.2016.00368

**Published:** 2016-03-21

**Authors:** Tanja G. Baudson

**Affiliations:** Institute of Psychology, Educational and Psychological Assessment, University of Duisburg-EssenEssen, Germany

**Keywords:** giftedness, harmony hypothesis, disharmony hypothesis, gifted stereotypes, social perception, big two, warmth and competence, stigma of giftedness

## Abstract

Scientists and laypeople agree on high ability as a defining feature of giftedness. Yet their views on gifted people's socioemotional characteristics diverge. Most studies find the gifted to be similar or slightly superior to average-ability persons in these domains (“harmony hypothesis”). However, subjective conceptions and media representations, most of which have focused on gifted children and youth, stress the socioemotional downsides of giftedness (“disharmony hypothesis”), affecting highly able individuals and those around them, thus hampering individual development. To date, most studies on gifted stereotypes have examined selective samples, mostly teachers. The present study is the first to provide representative data on conceptions of gifted individuals in general. A brief survey of 1029 German adults assessed quality and prevalence of stereotypes about gifted individuals, without an explicit focus on children and/or adolescents. Latent class analysis (LCA) revealed two conceptions of giftedness, with twice as many “disharmonious” than “harmonious” raters. Male gender, single parenthood, unemployment, higher income or negative attitudes toward the gifted predicted disharmonious ratings. However, effects were small, suggesting future studies look deeper into the processes of stereotype formation and maintenance.

## Introduction

Imagine two kids: one popular and well-adjusted, the other one uncomfortable around people and often unhappy. If you knew one of them were gifted—who would it rather be?

If you picked the sad loner, you are in good company. Individuals ascribe negative socioemotional characteristics to students described as gifted, such as isolation (Solano, 1987), lower agreeableness, higher introversion, and neuroticism (Baudson and Preckel, 2013). Although such stereotypes have little to do with actual characteristics of the gifted, they form a reality in people's minds and shape how they perceive and behave toward gifted individuals.

### Dimensions and conceptions of giftedness

When talking about giftedness and gifted individuals, we should keep in mind that there is no universally acknowledged definition of “gifted.” Giftedness is a social construct and thus depends on the cultural, historical, and social context it is used in. Furthermore, we should be aware that its definition is inseparable from the reason why we define it. Different dimensions of and approaches to defining giftedness will be outlined in the following.

#### Potential vs. achievement

In line with investment theory (e.g., Cattell, 1971), people differ in terms of their innate intellectual potential, or “fluid intelligence.” Similar to seed capital, potential can be “invested” into learning and the acquisition of so-called “crystallized intelligence,” e.g., knowledge (Schweizer and Koch, 2002). Because investment opportunities are related to age, children's giftedness is more likely to be defined in terms of potential, whereas gifted adults are rather judged in terms of their achievements.

#### Status vs. development

Time is but one factor of influence in talent development; and although potential and achievement are positively related, their correlation is by no means perfect. For instance, intelligence and grades correlate to about 0.50 (Jensen, 1998). Whether potential is fully developed into achievement depends on both intrapersonal and environmental catalysts, i.e., the personality characteristics and circumstances that support talent development (e.g., Gagné, 2004). Whereas static conceptions of giftedness pursue a “once gifted, always gifted” approach, developmental conceptions assume that giftedness may change over the lifespan—for the better or for the worse.

#### Uni- vs. multidimensionality

Giftedness may be defined by outstanding intellectual abilities only or include further dimensions (e.g., creative, psychomotor, or social abilities). However, most models of giftedness and talent agree on high intellectual ability as a characteristic feature of giftedness. Whereas early conceptions considered IQ measures to be sufficient to identify gifted children (e.g., Terman, 1925), more recent models of giftedness include other dimensions besides intellectual ability, too (e.g., Gagné, 2004).

#### Qualitative or quantitative differences

Empirical research suggests that the difference between “gifted” and “non-gifted” is quantitative rather than qualitative. Development of gifted students is accelerated, but follows similar trajectories as in average-ability students (e.g., Threlfall and Hargreaves, 2008). With respect to intellectual ability, common cut-off criteria such as “two standard deviations above the mean” are motivated statistically and not due to qualitative differences. In contrast, lay theories seem to perceive “the gifted” as a social group, thus as a distinct category, although this does justice neither to the rather quantitative differences between gifted and average-ability individuals nor to the heterogeneity of the gifted as a group (e.g., Achter et al., 1996).

In sum, the definition of giftedness depends on the purpose of this very definition. Whereas scientific theories strive to identify measurable characteristics of the gifted and integrate them into coherent theoretical frameworks, laypeople usually rely on subjective theories that are based on personal and media experiences with gifted individuals and may or may not be in line with scientific findings.

### Gifted stereotypes

The term “gifted” evokes stereotypes—images in our heads, based on beliefs about this group. In line with the above paragraph on qualitative vs. quantitative differences, Dai (2010) argues that rather than using sets of abstract characteristics to define and identify gifted individuals, people represent the gifted as prototypes. These include, but are not limited to, “high flyers” like Hermione Granger, who succeed at virtually anything; “mad geniuses” like Camille Claudel, whose sanity was the price she paid for being an outstanding sculptor; misunderstood “brilliant rebels” like Good Will Hunting, who do not actualize their potential (cf. Cox, 2000); or “geeks/nerds” like Sherlock Holmes, who are characterized by intellectual brilliance and complete lack (or disregard) of social abilities alike (see Cross, 2005, for a discussion of changing conceptions of geeks/nerds).

Across media types, the gifted are portrayed rather negatively, too. On television, they are underrepresented; generally, TV seems to provides few adequate role models for gifted children and adolescents (Abelman, 1992). A classic example from print is the “Sidis fallacy,” (Kett, 1978) “the wrongheaded, but widespread, idea that child prodigies grow into unproductive adults” (Bates, 2011, p. 375). Popular culture often portrays gifted students as nonathletic, unpopular, studying rather than having fun, and often female (Vialle, 2007). Contrarily, an analysis of the diachronic construction of giftedness in Estonian media showed that since the 2000s, giftedness is constructed as “the property of a product, a basis for success,” with a focus on individuals (particularly children) and sports, politics, or pop music (Põlda, 2015, p. 234). Apparently, Cox's (2000) recommendation to assume a critical stance of gifted students' media representations is perfectly justified.

Research on gifted stereotypes runs along two lines: the *disharmony hypothesis* (rooted in the “mad genius” myth; Becker, 1978; Gallagher, 1990; Neihart, 1999)—high ability implying deficits especially in socioemotional domains; and the *harmony hypothesis*, assuming in its strong form that gifted people excel at virtually anything (e.g., Terman, 1925; Mönks, 1963; Persson, 1998). Basically, these hypotheses can be broken down to two fundamental dimensions: one pertaining to potential, achievement, and related constructs, and one referring to socioemotional characteristics. Indeed, these two dimensions have been found to be crucial in intergroup perception. The Stereotype Content Model has termed these dimensions “warmth” and “competence” (Fiske et al., 2002, 2007; see Abele and Wojciszke, 2007, for further labels of this fundamental distinction). Warmth refers to people's intent, which can be either positive or negative, whereas competence refers to their ability to pursue this intent. Both dimensions are assumed to have developed in response to evolutionary pressures (Fiske et al., 2007). As the authors further point out, the two dimensions are likely to correlate moderately positively when individuals are judged, but may be independent in the judgment of groups. This corresponds well to the disharmony hypothesis, where the gifted are considered high in competence but low in warmth, thus representing an “envious stereotype” in terms of Fiske et al. (2002).

In sum, both harmony and disharmony hypothesis agree that gifted individuals are characterized by high potential and achievement (“competence”). Their distinguishing feature is their view on socioemotional abilities (“warmth”) of the gifted. Whereas the harmony hypothesis assumes similarity between gifted and average-ability individuals or even superiority of the gifted, the disharmony hypothesis conceives of the gifted as socially and emotionally inferior.

Gifted stereotypes are problematic because they shape gifted individuals self-perception. “Stigma of giftedness” theory posits that stereotyping may lead gifted students to either hide their potential to avoid identification with the stigma, or to overidentify with the label by adopting stereotypical characteristics (Coleman and Cross, 1988; Cross, 2005). However, stereotypes also affect others' perceptions and actions. For instance, Pajares (1992) showed that teachers' conceptions of gifted students shape expectations and, subsequently, students' classroom behavior and educational goals. Negative gifted stereotypes may therefore lead to potential remaining uncovered, underdeveloped, and misunderstood.

### Empirical findings about the gifted

But what does the empirical literature on actual differences between gifted and average-ability individuals say? As often, the truth lies somewhere in the middle, with a slant toward “harmony.” Regarding the “competence” dimension, we find that on average, gifted individuals are indeed superior in intellectual potential, achievement, and related characteristics. This includes better grades (Roznowski et al., 2000), greater openness to experience (DeYoung, 2011), higher academic self-concept and self-esteem (Roznowski et al., 2000), higher adaptive perfectionism (Parker, 1997; LoCicero and Ashby, 1999), lower performance anxiety (Richards et al., 2003), and higher educational aspirations (Roznowski et al., 2000). For the “warmth” dimension describing socioemotional characteristics, evidence favors great similarity between gifted and average-ability individuals. The gifted are no more prone to depression, anxiety, or suicide (Reis and Renzulli, 2004; Martin et al., 2010), show similar levels of wellbeing and stress (Zeidner and Shani-Zinovich, 2011), and are as agreeable as average-ability persons (Schilling, 2009; DeYoung, 2011), conscientiousness (Ackerman and Heggestad, 1997), and social abilities (Schilling, 2009; overview: Neihart et al., 2002).

### Summary and rationale for the present study

In sum, the gifted are characterized by (1) higher intellectual potential and (2), to a lesser extent, higher achievement. Differences favoring the gifted are usually too weak to suggest (3) their general superiority (e.g., Neihart et al., 2002). Contrary to media allegations, the gifted differ neither in (4) emotional stability nor (5) social relationships.

Research on gifted stereotypes suffers from nonrepresentative samples, often teachers (e.g., Endepohls-Ulpe and Ruf, 2005; Geake and Gross, 2008; Baudson and Preckel, 2013; Preckel et al., 2015). The “stigma of giftedness” (Coleman and Cross, 1988) is largely unexplored in gifted adults. Most of what we know about gifted stereotypes thus relates to teachers and students; the quality and prevalence of gifted stereotypes in representative samples are still unknown. Also, characteristics of people holding different stereotypes (e.g., “giftedness = socioemotional difficulties”) have not been identified, though they represent relevant starting points for changing misconceptions. One should also keep in mind that not only the vast majority of research, but also most of the counseling literature and most representations of the gifted in the popular media have focused on gifted children and adolescents, which may likely influence people's conceptions of giftedness.

With these questions in mind, I surveyed a representative German sample (1029 adults) about their ideas about the gifted. A brief questionnaire included five core aspects of gifted stereotypes: (1) higher potential, (2) higher achievement, (3) general superiority, (4) emotional problems, and (5) social issues. Latent class analysis (LCA) differentiated groups with characteristic “rater profiles” by maximizing both homogeneity within and heterogeneity between groups. Theory suggested two profiles: “harmonious” vs. “disharmonious,” differing in ratings of emotional and social problems, but comparable in potential, achievement, and general superiority ratings, with a possible third “extremely harmonious” profile assuming general superiority in the gifted. Logistic regression identified rater characteristics (i.e., statistical predictors of latent class membership), examined exploratorily for lack of relevant research.

## Methods

### Sample

The sample was representative with respect to age, gender, and regional distribution for German adults between 18 and 69 years of age. Data were collected with the help of INNOFACT AG, a provider specialized in representative surveys. Altogether, 1029 adults (50.5% female; mean age = 43.85 years, *SD* = 14.31) took part in the study.

### Materials

The questionnaire included 10 demographical items about the participants' background: gender, age, federal country of Germany the participants lived in, level of education, current occupational status, monthly net income, family status, and household size, including information about children under 18. The remaining nine questions addressed opinions about gifted persons along the five core dimensions described above: intellectual potential, achievement, social difficulties, emotional issues, and superiority in other domains beside intellect (5 items, rated on 5-point Likert scales from “do not agree at all” to “absolutely agree”). Furthermore, participants rated their own intelligence (5-point scale from “substantially below average” to “substantially above average”), the feelings the term “giftedness” evoked in them (4-point scale from “very negative” to “very positive”), their interest in giftedness (4-point scale from “not interested at all” to “very interested”), and whether they knew any gifted persons (1 item each). The option “cannot/do not want to answer” was provided with all items.

### Procedure

The 10 demographical items were a standard part of the omnibus survey. The nine giftedness-related questions were compiled by the author and the strategic development team of the high-IQ society Mensa in Germany. Data were collected as part of the weekly online omnibus survey of a marketing research institute specialized in these services. The survey was funded by Mensa in Germany as part of their strategic development and their efforts to support scientific research on giftedness. The author analyzed the data using SPSS 22.0.0.1 (IBM Corp., 2013; descriptives) and Mplus 7.11 (Muthén and Muthén, 1998–2012; latent class analyses).

Because no clear-cut criteria have yet been proposed to decide on the number of latent classes, a combination of indices was used (Nylund et al., 2007; Geiser, 2011). Criteria included (1) theoretical soundness, the simplest theoretically sound solution being two classes, namely, the “harmonious” vs. the “disharmonious” view; (2) parsimony, which would exclude solutions with highly similar classes; (3) average classification probability, which should exceed 0.80 (Geiser, 2011); (4) entropy (a global measure of how reliable the classification is, 1.00 being the maximum), (5) information criteria such as AIC, BIC, and sample-size adjusted BIC; and (6) statistical tests assessing whether increasing the number of classes improves fit. Here, the Vuong-Lo-Mendell-Rubin Likelihood Ratio Test (VLMR LRT), the Lo-Mendell-Rubin adjusted Likelihood Ratio Test (LMR LRT) and the Bootstrapped Likelihood Ratio Test (BLRT) were used, which allow for a direct comparison between neighboring solutions (*k* vs *k*-1 classes).

## Results

### Descriptives

Descriptives are given in Table 1. Overall, participants rated gifted persons as somewhat more positive than negative. However, correlations (Table 2) showed that positively (potential, achievement) and negatively connoted characteristics (social and emotional issues) are positively (though weakly) correlated.

**Table 1:** **Gifted ratings and attitudes toward giftedness**.

			**Range**	
**Variable**	***M***	***SD***	**Theoretical**	**Empirical**
**GIFTED RATINGS**				
(from 1 = *do not agree at all* to 5 = *absolutely agree*)				
– Higher potential “Gifted individuals have a higher intellectual potential than average-ability individuals”	03.94	0.94	1–5	1–5
– Higher achievement “Gifted individuals achieve at higher levels than average-ability individuals (e.g., better grades, higher educational levels)”	03.43	1.06	1–5	1–5
– General superiority “Gifted individuals are superior to average-ability individuals even in domains not directly related to intellectual abilities”	02.68	1.03	1–5	1–5
– Emotional issues “Gifted individuals are more likely to have emotional issues than average-ability individuals.”	03.60	0.98	1–5	1–5
– Social difficulties “Gifted individuals are more difficult in social interactions than average-ability individuals.”	03.56	0.99	1–5	1–5
**ATTITUDES TOWARD GIFTEDNESS**				
– Positive associations “When hearing the word “gifted,” what emotions does the term evoke in you? “ (from 1 = *very negative* to 5 = *very positive*)	03.38	0.87	1–5	1–5
– Interest “Are you interested in giftedness?” (from 1 = *not at all* to 4 = *very much*)	02.84	0.79	1–4	1–4

Higher ratings indicate higher agreement with the variable in question. The original German items are available from the author.

**Table 2:** **Intercorrelations amongst respondents' ratings of the gifted (single-item measures)**.

	**1**	**2**	**3**	**4**
1. Higher potential	–			
2. Higher achievement	0.34^***^	–		
3. General superiority	0.11^**^	0.20^***^	–	
4. Emotional issues	0.14^***^	0.08^*^	0.09^**^	–
5. Social difficulties	0.14^***^	0.08^*^	0.06^+^	0.53^***^

+ *p < 0.10*.

* *p < 0.05*.

** *p < 0.01*.

*** *p < 0.001*.

### Latent-class analysis

**Figure 1:**
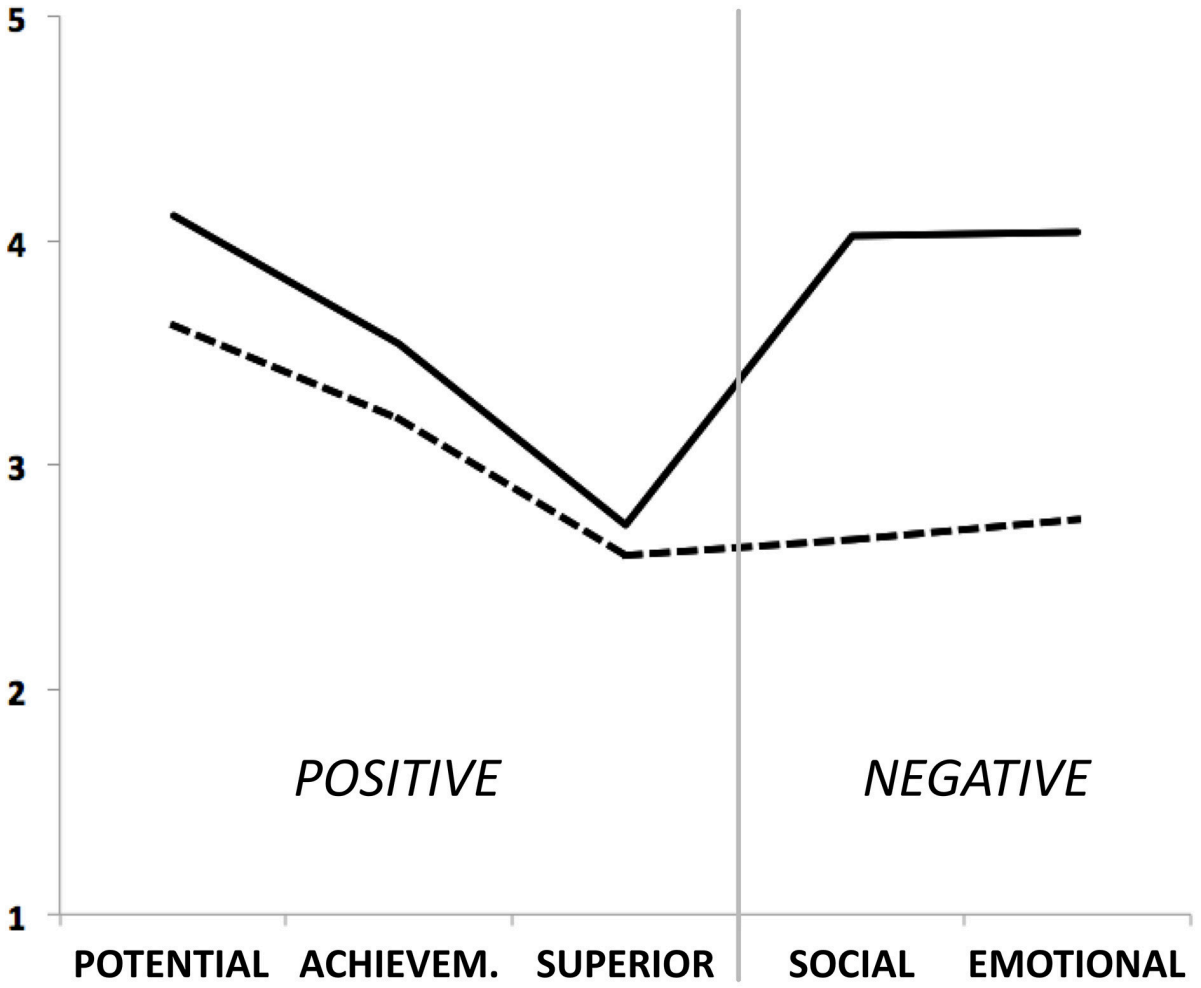
**“Harmonious” and “disharmonious” stereotypes**. Rater profiles of the “harmonious” class (dotted line), which ascribes high potential, high achievement, no pronounced superiority and average ratings on social and emotional issues to gifted persons, and the “disharmonious” class (solid line), which rates gifted persons similar in potential, achievement, and superiority, but high in negative social and emotional characteristics.

To examine whether this pattern held across the entire sample, or whether different subgroups of raters should be differentiated, an LCA was performed. Table 3 provides an overview of the fit indices for solutions with different numbers of classes. With respect to theoretical foundation (harmony vs. disharmony hypothesis), parsimony and distinctness of classes, average probability of correct classification, and results of the statistical tests (VLMR, LMR, and BLRT), a two-class solution was deemed to fit the data best. Also, the decline in relative fit indices (AIC, BIC, adjusted BIC) was sharpest between the one- and the two-class solution. Entropy was the only criterion disfavoring the two-class solution. Figure 1 provides a graphical representation of the average ratings for the two-class solution.

**Table 3:** **Latent class analysis results**.

	**Number of latent classes**				
	**1**	**2**	**3**	**4**	**5**
Average LC probability	1.00	0.88	0.82	0.82	0.80
Entropy	–	0.63	0.68	0.68	0.71
**INFORMATION CRITERIA**					
– AIC	13,934.65	13,630.85	13,526.59	13,467.56	13,412.41
– BIC	13,983.85	13,709.57	13,633.81	13,605.31	13,579.68
– adjusted BIC	13,952.09	13,658.75	13,563.95	13,516.38	13,471.69
**COMPARATIVE INDICES^*a*^**					
– VLMR LRT	–	*p* < 0.0001	*p* = 0.33	*p* = 0.19	*p* = 0.31
– LMR LRT	–	*p* < 0.0001	*p* = 0.33	*p* = 0.19	*p* = 0.31
– Bootstrapping LRT	–	*p* < 0.0001	*p* < 0.0001	*p* < 0.0001	*p* < 0.0001

*LC, latent class; VLMR LRT, Vuong-Lo-Mendell-Rubin Likelihood Ratio Test; LMR LRT, Lo-Mendell-Rubin Likelihood Ratio Test*.

a *Comparison of neighboring solutions (k vs. k-1 classes)*.

### Predictors of class membership

I further examined which variables predict class membership using binary logistic regression (Table 4). Men were 1.39 times more likely to attribute a “disharmonious” profile (i.e., greater social and emotional issues) to gifted individuals. While age had no influence, family status did: Compared to singles without kids, single parents were more than three times more likely to perceive the gifted in line with the disharmony hypothesis. With respect to the raters' educational and professional situation, unemployed individuals were 2.81 times more likely to associate giftedness with the disharmonious profile, compared to raters employed fulltime. Except for the highest income group (≥3800 €), the likelihood of rating gifted persons as disharmonious increased with income, but only reached significance in the 2500–3800 € group. In contrast, educational level had a marginal influence only. Of the giftedness-related variables, only positive emotions toward giftedness (about 21% lower probability of disharmonious ratings per step in the more “positive” direction) predicted class membership, whereas interest in giftedness was tendentially related to a greater probability of disharmonious ratings (*OR* = 1.23, *p* = 0.06). Neither knowing a gifted person nor self-rated intelligence level had any significant influence.

**Table 4:** **Binary logistic regression results—predictors of group membership (0 = “harmonious,” 1 = “disharmonious” raters)**.

					**95% CI**	
	***B* (*SE*)**	**Wald (*df*)**	***p***	***OR***	**Lower**	**Upper**
Intercept	−0.02 (1.54)	0.00 (1)	0.99	0.98	0.99	1.02
**STEP 1: DEMOGRAPHICS (*R*^2^ = 0.01/0.02)**						
Gender (0 = *male*, 1 = *female*)	−0.33 (0.16)^*^	4.16 (1)	0.04	0.72	0.52	0.99
Age	−0.00 (0.01)	0.00 (1)	0.98	1.00	0.99	1.02
Family status (reference group: single, no kids)		6.84 (3)	0.08			
– With partner, no kids	−0.23 (0.19)	1.52 (1)	0.22	1.26	0.87	1.80
– Single with kid	−1.13 (0.45)^*^	6.35 (1)	0.01	3.11	1.29	7.51
– With partner and kid(s)	−0.18 (0.24)	0.60 (1)	0.44	1.20	0.76	1.91
**STEP 2: EDUCATIONAL AND PROFESSIONAL SITUATION (*R*^2^ = 0.03/0.05)**						
Educational level (reference group: lowest school track, no professional training)		3.34 (4)	0.50			
– Lowest track, professional training	−0.78 (0.43)^+^	3.23 (1)	0.07	2.18	0.93	5.12
– Intermediate track	−0.69 (0.42)	2.65 (1)	0.10	2.00	0.87	4.58
– Academic track, no tertiary education	−0.73 (0.43)^+^	2.80 (1)	0.09	2.06	0.88	4.82
– Academic track, tertiary education	−0.70 (0.44)	2.58 (1)	0.11	2.01	0.86	4.73
Professional situation (reference group: working full time)		8.45 (5)	0.13			
– Part time	−0.08 (0.23)	0.12 (1)	0.73	1.08	0.69	1.69
– Unemployed	−1.03 (0.38)^**^	7.59 (1)	0.01	2.81	1.35	5.85
– Retired	−0.13 (0.26)	0.23 (1)	0.63	1.14	0.68	1.90
– Homemaker	−0.12 (0.33)	0.14 (1)	0.71	1.13	0.59	2.18
– Student	−0.43 (0.33)	1.75 (1)	0.19	1.54	0.81	2.91
Household net income (€/month; reference group: < 1,000 €)		6.22 (5)	0.29			
– 1000 ≤ income < 1500 €	−0.24 (0.27)	0.81 (1)	0.37	1.27	0.76	2.13
– 1500 ≤ income < 2000 €	−0.52 (0.28)^+^	3.38 (1)	0.07	1.69	0.97	2.94
– 2000 ≤ income < 2500 €	−0.55 (0.29)^+^	3.71 (1)	0.05	1.73	0.99	3.04
– 2500 ≤ income < 3800 €	−0.56 (0.28)^*^	3.93 (1)	0.05	1.75	0.99	3.04
– ≥3800 €	−0.32 (0.31)	1.07 (1)	0.30	1.38	0.75	2.53
**STEP 3: GIFTEDNESS**−**RELATED VARIABLES (*R*^2^ = 0.04/0.06)**						
Knowing a gifted person (reference group: no)		0.07 (2)	0.97			
–Maybe	−0.03 (0.20)	0.02 (1)	0.88	1.03	0.70	1.53
– Yes	−0.06 (0.22)	0.07 (1)	0.79	1.06	0.69	1.64
Interest in giftedness	−0.21 (0.11)^+^	3.65 (1)	0.06	1.23	1.00	1.53
Positive emotions toward giftedness	−0.23 (0.09)^*^	6.05 (1)	0.01	0.79	0.66	0.95
Self−rated IQ level (reference group: IQ < 70)		1.69 (4)	0.79			
– 70 ≤ IQ < 85	−0.57 (1.66)	0.12 (1)	0.73	0.57	0.02	14.64
– 85 ≤ IQ < 115	−0.23 (1.52)	0.02 (1)	0.88	0.79	0.04	15.73
– 115 ≤ IQ < 130	−0.31 (1.52)	0.04 (1)	0.84	0.73	0.04	14.55
– IQ ≥ 130	−0.64 (1.55)	0.17 (1)	0.68	0.53	0.03	11.08

*OR, odds ratio; R^2^, pseudo R^2^ (Cox and Snell/Nagelkerke)*.

+ *p < 0.10*.

* *p < 0.05*.

** *p < 0.01*.

Classification accuracy improved little through stepwise inclusion of the predictors. The null model classified 67.5% of all cases correctly, which did not change when demographical characteristics were included, and increased only by 0.3% after inclusion of professional background variables. Giftedness-related variables did not impact the absolute classification accuracy, yet led to more “harmonious” raters being classified correctly. Absolute figures were still small, though. The changes in percentage with each regression step were 0/0/3.9/7.0% for the “harmonious” group, compared to 100/100/98.5/97% for the “disharmonious” group. The Hosmer-Lemeshow test exceeded the 10% probability level in all cases, suggesting that the null hypothesis (i.e., the model fits the data) should not be discarded.

## Discussion

### Summary of the findings

Conceptions of giftedness come in two shapes: *harmony*, characterized by high potential and achievement ratings, and *disharmony*, comparable in potential, achievement, and general superiority ratings, but ascribing more social and emotional problems to the gifted. This study provides the first representative picture of how people in a Western society conceive of the gifted. The survey covered five crucial aspects of gifted stereotypes. Partially, results align with research revealing disharmonious conceptions of giftedness in teachers (Baudson and Preckel, 2013; Preckel et al., 2015). However, this is the first study to quantify both negative *and* positive stereotypes, showing that 2/3 of the respondents hold a negative stereotype. Though some demographic and psychological predictors were identified, much variance remained unexplained.

### Bakan's “duality of human existence” revisited

It seems surprising that gifted stereotypes are no more complex than this. However, their underlying structure aligns perfectly with Fiske et al.'s (2007) warmth vs. competence dimensions of intergroup perception. As mentioned above, the disharmonious gifted stereotype would represent an “envious” stereotype, comparable to other highly able, but cold groups who have the capacity to pursue their intent, but whose intent is not necessarily in line with the community's (e.g., career women or Germans; Fiske et al., 2002). In contrast, the harmonious gifted stereotype, which is characterized by high competence and at least “normal” warmth, might be considered a reference group, a “normative standard for social comparison and most often, social aspiration” (Cuddy et al., 2009, p. 6). In individualistic societies like Germany, these reference groups are usually located in the “competent and warm” quadrant, which would fit the results presented here.

As participants were supposed to rate the gifted on five characteristics relating to gifted stereotypes, the findings can also be related to the “Big Two” of personality: agency (here: potential/achievement) and communion (here: socioemotional issues, or lack thereof; Bakan, 1966). The Big Five forming these two “superfactors” are also reflected directly in the items characterizing the gifted. For agency, openness (the dimension most strongly associated with intelligence) may be considered part of “higher potential,” whereas “higher achievement” comprises conscientiousness, success usually requiring effort. For community, neuroticism and agreeableness/extraversion are represented by proneness to emotional issues and social difficulties, respectively^1^.

A “strongly harmonious” gifted stereotype including general superiority (Terman, 1925) was not identified, suggesting that a realistic image of high competence and at least average warmth is as good as it can get for the gifted. Because the gifted are indeed superior in some agentic aspects (potential, achievement), Paulhus and John's (1998) “superhero bias” (i.e., exaggerated self-ascribed agency) is not reflected in gifted stereotypes. There is no evidence of any “saint” bias either, as neither of the two gifted stereotypes comprises superior community.

### Understanding predictors of gifted stereotypes

The low variance explained suggests that future studies should look deeper into the mechanisms of stereotyping. E.g., people intimidated by the gifted's undisputably higher potential might devalue their (imaginary) competitors by ascribing them negative socioemotional characteristics, thus coping with perceived threat and inferiority, which might explain why unemployment predicts disharmonious ratings. That the same relationship was identified for males and higher income groups corroborates this interpretation: Genders respond differently to intergroup threat and competition (Van Vugt et al., 2007; Sutter and Rützler, 2010). This fits well into the two-dimensional frameworks of self- and other-perception outlined above. Competitiveness, intergroup/ego threat, perceived injustice, envy, and sense of entitlement are therefore promising predictors to examine in future studies. Considering that the “envious” stereotype may also be a consequence of perceiving others as both highly competent and cold, it might also be interesting to examine possible reciprocal effects between stereotyping of and emotions toward certain groups longitudinally, hereby also differentiating different emotions beyond the rather coarse positive/negative associations with the term used here.

Knowing a gifted person did not influence whether a participant rated the gifted as harmonious or disharmonious. This is notable with respect to the slight positive halo effect Fiske et al. (2007) report for the judgment of individuals and allows for diverse interpretations. It is possible that respondents who know “harmonious” and “disharmonious” gifted individuals are about evenly distributed, thus canceling each other out. An alternative explanation is that despite the greater visibility of disharmonious gifted individuals, raters are nevertheless able to make a balanced judgment and not overgeneralize their personal experiences. Future studies may want to look deeper into respondents' actual experiences with gifted individuals to identify differential effects.

Single parents more readily ascribe socioemotional difficulties to the gifted. Children of divorce are less adjusted (meta-analyses: Amato and Keith, 1991; Amato, 2001), probably due to conflicts preceding divorce (Hetherington, 1999). Additionally, parents overrate their offspring's intelligence (e.g., Miller et al., 1991). Speculatively, parents might misattribute socioemotional problems to their children's (assumed) giftedness, a question worth pursuing.

Self-rated intelligence was unrelated to latent class membership, suggesting highly intelligent persons (= gifted by IQ) are no more likely to see their ingroup as overproportionally positive. In line with the stereotype content model, this allows for at least two interpretations. First, harmonious giftedness might be an ideal to aspire to, but the harmoniously gifted may not be considered part of one's ingroup. Second, it is conceivable that though high intelligence is a characteristic of both implicit and explicit theories of giftedness (e.g., Baudson and Preckel, 2013), it is not sufficient to define giftedness, such that even highly intelligent raters would not necessarily identify themselves as gifted. Possibly, this is due to the stigma of giftedness: Highly intelligent individuals might refuse the label “gifted” because a large majority associates the term with negative characteristics. This has interesting implications for identity development in minorities (e.g., Frable, 1997), a topic yet underexamined in giftedness research.

Though largely representative, our data span Germany only, which likely impacts the content of stereotypes (e.g., Cuddy et al., 2009). Also, the value of effort vs. innate ability (Dweck, 2007) or the representation of gifted programs in the media (Karnes and Lewis, 1995) may affect both quality and quantity of gifted stereotypes internationally. Research should address these questions, also to develop culture-sensitive interventions.

### Calling for action

As all countries scarce in natural resources, Germany depends on brainpower. Yet many Western countries fail to cater for their most able students' educational needs (Colangelo et al., 2004; Assouline et al., 2015). One reason may be that two thirds of the population perceive the gifted as socioemotionally problematic, possibly even dangerous to the community when low social orientation meets high intelligence (Geake and Gross, 2008). Possibly, fostering the gifted is perceived as unfair in two ways: because the gifted would be able to help themselves (high ascribed potential and achievement); and because later benefits for society are uncertain (low ascribed community). This question touches on fundamental social values—who deserves support, and who does not?—, which should be understood and clarified interdisciplinarily before taking action.

Stereotypes shape the perception of reality. That negative gifted stereotypes are twice as common as realistic ones makes the disharmonious “outgroup” perspective (likely acquired through others passing on the stereotype) as comprehensible as the “ingroup”'s reluctance to label themselves as gifted. The negative gifted stereotype's ambivalence (amalgamating positive and negative ascriptions) should therefore be covered when addressing the gifted's socioemotional needs (e.g., Peterson, 2008).

Stereotypes may also shape actions. Misconceptions of giftedness should therefore be remedied (which is hard, but not impossible; e.g., Schack and Starko, 1990; Hoogeveen et al., 2005). Mere exposure is unlikely to succeed: Knowing gifted persons did not impact people's conceptions of giftedness here. A deeper involvement tackling the underlying dynamics of stereotyping thus seems more promising.

Popular media creating and perpetuating stereotypes should use their power responsibly, even if this clashes with economic interests. Scientists should enter the public discourse to counterbalance misrepresentations critically.

## Conclusion

Accurate knowledge about giftedness may translate into greater awareness, hint at possible remedies, and eventually improve conditions for our most talented citizens. The challenge to convey this knowledge should be accepted concertedly by researchers, practitioners, and the media. Keeping high ability and socioemotional deficits apart would help gifted people accept and actualize their potential—for their own sake and for that of a caring and supportive society.

## Author contributions

TGB provided the theoretical background, developed major parts of the questionnaire, analyzed the data and wrote the paper.

## Funding

Data collection was funded by Mensa in Germany, e. V.

### Conflict of interest statement

The author declares that the research was conducted in the absence of any commercial or financial relationships that could be construed as a potential conflict of interest.

## Footnotes

^1^Considering the generality of these two core dimensions, it is conceivable that the incompatibility between high potential and success on the one hand and socioemotional difficulties on the other is not limited to the gifted, but possibly extends to high achievers in general, as one reviewer pointed out. As such, the stereotype might be interpreted as a general human desire for fairness. The design of our study does not allow to rule out this possibility. However, I would like to offer a few speculative thoughts on why giftedness may more than mere high ability in intergroup perception. First, not all high abilities are created equal. As Geake and Gross (2008) point out, psychomotor prowess and musical abilities are valued because of their direct usefulness to the group. In contrast, high verbal ability has the potential to destroy the existing order, especially when coupled with low regard for others. But even if we limit “high ability” to “high intellectual ability,”, the qualifier “high” suggests that a highly able person is to be found somewhere in the upper tail of the ability continuum. One would rather speak of low achievement or intelligence than of ^*^non-achievement or ^*^non-intelligence. In contrast, “gifted” versus “non-gifted” or “average-ability” persons would rather suggest a categorical distinction.
